# Understanding Solidification of Polythiophene Thin Films during Spin-Coating: Effects of Spin-Coating Time and Processing Additives

**DOI:** 10.1038/srep13288

**Published:** 2015-08-24

**Authors:** Jin Yeong Na, Boseok Kang, Dong Hun Sin, Kilwon Cho, Yeong Don Park

**Affiliations:** 1 Department of Energy and Chemical Engineering, Incheon National University, Incheon 406-772, Korea; 2 Department of Chemical Engineering, Pohang University of Science and Technology, Pohang 790-784, Korea

## Abstract

Spin-coating has been used extensively in the fabrication of electronic devices; however, the effects of the processing parameters have not been fully explored. Here, we systematically characterize the effects of the spin-coating time on the microstructure evolution during semiconducting polymer solidification in an effort to establish the relationship between this parameter and the performances of the resulting polymer field-effect transistors (FETs). We found that a short spin-coating time of a few seconds dramatically improve the morphology and molecular order in a conjugated polymer thin film because the π-π stacking structures formed by the polymer molecules grow slowly and with a greater degree of order due to the residual solvent present in the wet film. The improved ordering is correlated with improved charge carrier transport in the FETs prepared from these films. We also demonstrated the effects of various processing additives on the resulting FET characteristics as well as on the film drying behavior during spin-coating. The physical properties of the additives are found to affect the film drying process and the resulting device performance.

Spin-coating techniques are remarkably easy to use, versatile, inexpensive, and highly effective for depositing a uniform thin film reproducibly^1^,^2^,^3^. As a result, the technique has been extensively used to fabricate solution-processed organic/inorganic electronic devices^4^,^5^,^6^. Organic semiconductors have been spin-coated onto substrates to fabricate high-performance organic field-effect transistors (OFETs) that have attracted particular attention as key elements for realizing next-generation flexible printed electronics^7^,^8^,^9^,^10^,^11^,^12^. The performances of OFETs prepared by spin-coating are strongly correlated with the morphology and solid-state order of the deposited semiconducting layer^13^,^14^, and these properties depend significantly on the processing conditions applied during spin-coating, such as the solvent properties^15^,^16^, the substrate temperature^17^,^18^,^19^ and surface characteristics^20^,^21^, the ambient solvent vapor pressure^22^, and the spinning speed^23^,^24^ or time. The careful adjustment of all processing parameters can maximize the performance of the OFETs fabricated by spin-coating, given the inherent limitations of the semiconducting material.

Many mathematical models of the spin-coating process have been developed to predict the thickness as a function of the various processing parameters to a high degree of accuracy^25^,^26^. The molecular-scale structure and properties of the resulting film, on the other hand, cannot be readily predicted because the spin-coating process itself is highly complicated due to the many mechanisms involved^27^. The effects of the processing conditions on the performances of OFETs, for example, must be tested empirically. The spinning speed may be tuned to alter the strength of the centrifugal force and shear force, thereby varying the final film thickness as well as the film roughness and crystalline structures^23^,^24^. These features in turn affect the charge injection and transport properties in the OFETs^28^,^29^. Distinct behaviors may be obtained, depending on the spin speed, the materials, and the conditions. The choice of solvent used during the fabrication of OFETs can also affect the OFET performance^15^,^30^. A slow solvent evaporation rate allows time for the polymer chains to self-organize, thereby facilitating the growth of a highly crystalline film. The use of high boiling point solvents, however, is limited to substrates with a high surface energy and does not always yield a high charge mobility in the OFETs. These effects result from de-wetting of the thin film, poor crystalline connectivity, and charge trapping from the residual solvent^30^. Nevertheless, rules of thumb would be a significant help to researchers seeking to characterize the processing–structure–property–performance relationship^31^,^32^. Additional efforts to elucidate this relationship could improve the performances of OFETs and contribute to the commercialization of OFET-based highly integrated circuits and devices.

Little is known about the effects of the spin-coating time (the “spinning time”) on OFET performance. In the majority of reports describing the fabrication of solution-processed OFETs, the spinning time ranges from 40s to 180s, depending on the volatility of the solvents^8^,^15^,^33^,^34^. A conductive polymer was spin-coated for a brief time of a few seconds by Lee *et al.*^35^. Doped polyaniline films with various thicknesses were produced by controlling the spin speed for a fixed spinning time of 3s. The resultant polyaniline thin films showed high thickness-independent conductivities, which were attributed to the improved degree of crystallinity. These results gave us a vital clue about the relationship between the spinning time and the OFET performance. In this paper, we systematically investigated, for the first time, the effects of the spinning time on the microstructure evolution in polymer thin films to establish the relationship between the polymer FET device performances. To this end, the morphologies, microstructures, and optical and electrical properties of the semiconducting polymer thin films were thoroughly investigated using atomic force microscopy (AFM), two-dimensional grazing-incidence X-ray diffraction (2D GIXD), UV-vis spectroscopy, and FET device measurements. The spin-coating process was optically monitored to obtain important information about the drying behavior of the solution during spin-coating and to help explain why the brief spinning time improved the charge transport characteristics of the semiconducting polymer films. In the section that follows, we discuss our attempts to control the drying behavior of a solution by including the processing additives, which significantly altered the polymer thin film solidification time. Finally, polymer FETs processed with various additives for a given spinning time were characterized, revealing that the effective spinning time range could be controlled using processing additives.

## Results

We examined the most widely studied semiconducting polymer, poly(3-hexylthiophene) (P3HT), as a reference for this initial investigation. Chlorobenzene (CB) was chosen as the solvent because its high boiling point (131 °C) made available a wide window of spin-coating times. A solution comprising P3HT in CB was spin-coated onto hexamethyldisilazane (HMDS)–treated SiO_2_/Si substrates or glass substrates for different spinning times (3, 5, 10, 30, and 60s, respectively)^20^. The samples were then held in a vacuum to completely dry the solvent. First of all, we examined the UV-Vis absorption spectrum obtained from the P3HT solution. The solution spectrum contained only one peak at *λ* = 455 nm associated with the intra-chain π-π* transition of P3HT. No signs of molecular ordering were observed, indicating that the P3HT chains remained molecularly well-dissolved as in the bulk CB solution state (see Figure S1 in the Supporting Information).

**Figure 1:**
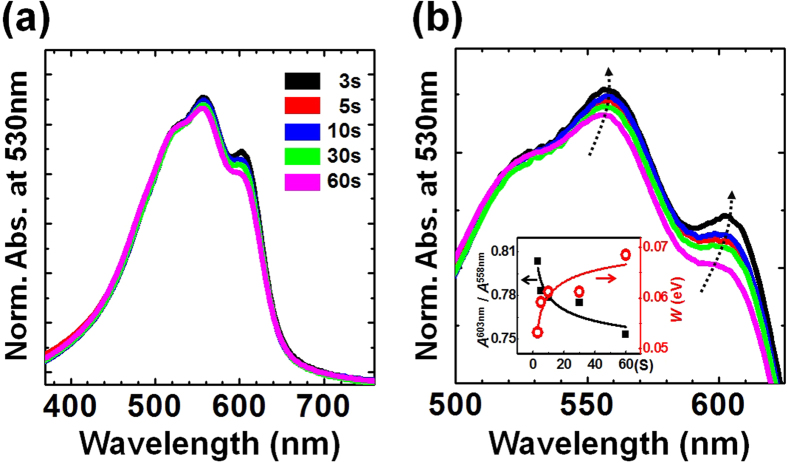
UV-Vis absorption spectral analysis. (**a**) UV-Vis absorption spectra showing the normalized absorption bands at the 0–2 transition (*λ* = 530 nm) in the P3HT thin films spin-cast for various spinning times. (**b**) Magnified view of the normalized UV-Vis absorption bands. The spinning time applied during the spin-coating process decreased along the direction of the arrow. The inset shows the intensity ratio between the first (*λ* = 603 nm) and second (*λ* = 558 nm) vibronic transitions and the interchain coupling energy *W* as a function of the spinning time.

Figure 1 shows the normalized UV-Vis absorption spectra of the P3HT thin films spin-coated for various spinning times. The spectra obtained from the P3HT thin films displayed a dominant peak at *λ* = 530 nm corresponding to an intrachain π-π* transition of P3HT, with two minor shoulders at lower energies (*λ* = 558 nm and 603 nm), indicating interchain π-π stacking interactions (Fig. 1a)^36^,^37^. As the spinning time was reduced from 60s to 3s, the intensities of the two shoulder peaks gradually increased (Fig. 1b). The absorption and emission spectra of lamellar organized aggregates of P3HT can be described and understood in terms of an H-aggregate type chromophore with weak interchain coupling interactions. In this model, the intensity of the first transition (0−0) in the absorption spectrum (*λ* = 603 nm) is reduced by increasing the excitonic coupling. The magnitude of the interchain coupling energy W can be extracted from the intensity ratio of the first and second vibronic transitions (0−1) in the thin film absorption spectrum using the following equation (see the inset shown in Fig. 1b)^37^,


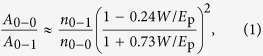


where *n*_0−1_ is the refractive index at the (0−1) peak and *E*_p_ is the vibration energy coupled to the electronic transition (0.18 eV for P3HT). The refractive index ratio *n*_0−1_/*n*_0−0_ is nearly equal to 1 (0.97 for a P3HT thin film)^38^. The calculated interchain coupling energy *W* decreased from 0.069 to 0.053 eV at short spinning times. A low interchain coupling energy is associated with longer conjugation lengths produced by the high crystalline order in the thin films^39^. Therefore, the appearance of features in the UV-vis spectra indicated that shorter spinning times induced the development of a larger number of ordered P3HT aggregates containing interchain π-π stacking interactions in the films.

**Figure 2:**
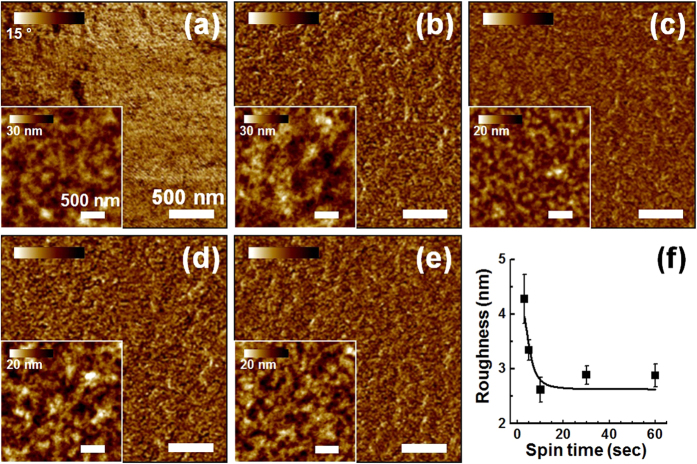
Morphological analysis. AFM phase images of the P3HT films that had been spin-cast for various spinning times: (**a**) 3s, (**b**) 5s, (**c**) 10s, (**d**) 30s, and (**e**) 60s. The inset shows the height images of each film. (f) The root-mean-square (RMS) roughness of the film surface is provided.

These results agreed well with the morphological changes observed in the P3HT films (Fig. 2a–e). Unlike the nanoscale aggregated features of the P3HT films cast for 10–60s, the AFM phase images of the P3HT films cast over 3–5s revealed nanofibrillar network structures that would provide efficient charge transport pathways^32^. Moreover, the root-mean-square (RMS) surface roughness values of the thin films tended to increase at shorter spinning times (Fig. 2f). These results supported the improved crystallinity of the P3HT film spin-cast over a short spinning time compared to the crystallinity of the P3HT films cast over 60s^40^. The UV-vis spectra and morphologies revealed that a shorter spinning time could effectively induce intermolecular π-π stacking among the polymer chains during the spin-coating process.

**Figure 3:**
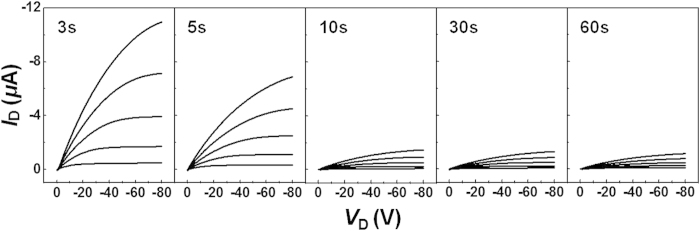
*I*-*V* characteristics of polythiophene thin films adopting a transistor configuration. (**a**) Output curves (*I*_D_ − *V*_D_) of the FETs (*V*_G_ steps = 0, −20, −40, −60, and −80 V) fabricated using P3HT thin films that had been spin-cast for various spinning times.

**Figure 4:**
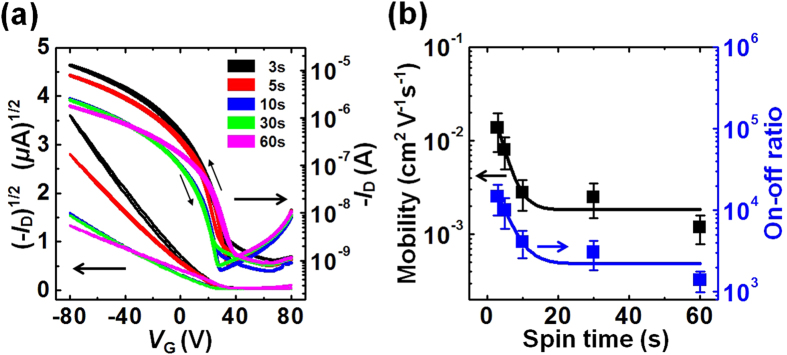
Detailed electrical characteristics and summary of device performance. (**a**) Transfer characteristics (*I*_D_ − *V*_G_) of the FETs (*V*_D_ = −80 V) fabricated using P3HT thin films that had been spin-cast for various spinning times. (**b**) Field-effect mobilities (left axis) and on-off ratios (right axis) obtained from the P3HT FETs as a function of the spinning time.

The effects of the spinning time on the electrical properties (the *I*-*V* characteristics) of the OFETs prepared based on the P3HT films were systematically characterized (see the Methods section for details). All devices displayed well-behaved p-channel operation with ohmic behaviors in the linear region of the output characteristics, as shown in Fig. 3 (low drain-source voltages (*V*_D_)). The on-current levels (*I*_D_ at *V*_G_ = −80 V) gradually decreased and reached saturation in the devices prepared with a spinning time of 10s, about one order of magnitude lower than the on-current levels measured in FETs prepared using the P3HT films cast over 3s. This trend was similar to the roughness increase observed in the morphology measurements (Fig. 2f). The charge transport properties of the P3HT films were further characterized by measuring the transfer characteristics (Fig. 4a). The transfer curves showed negligible hysteresis during forward and backward sweeps, implying that the solvent was completely dried. The FETs prepared with P3HT cast for 3s displayed the highest mobility value of 0.014 cm^2^ V^−1^ s^−1^ among the samples, more than ten times the value of the P3HT films cast for 60s. The mobility values gradually decreased and reached saturating values in the device prepared with longer spinning times (Fig. 4b). It should be noted that the high mobility values were achieved in FETs prepared using P3HT thin films cast for 3–5s without post-treatments, such as thermal annealing or solvent annealing. However, compared to the previously reported values^41^,^42^, the mobility of 0.014 cm^2^ V^−1^ s^−1^ in our experiments is somewhat low, which might be due to low regioregularity (~95%) of the P3HT.

Furthermore, the sub-threshold slope of the devices showed an increasing tendency as the spinning time decreased. This trend can be used to measure the maximum interfacial trap density using the following equation^43^:


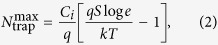


where *q* is the electronic charge, *S* is the sub-threshold slope (V decade^−1^), *k* is the Boltzmann constant, and *C*_i_ is the specific capacitance of the gate dielectric. Because *S* decreased from 10.0 (for a spinning time of 60s) to 6.7 V (for a spinning time of 3s), the calculated trap density decreased from 1.12 × 10^13^ to 7.54 × 10^12^ cm^−2^ eV^−1^. This reduction could be attributed to the presence of fewer trapping sites at the semiconductor–dielectric interface (or at the bulk semiconductor layer) as a result of the improved self-organization of P3HT prepared over brief spinning times.

**Figure 5:**
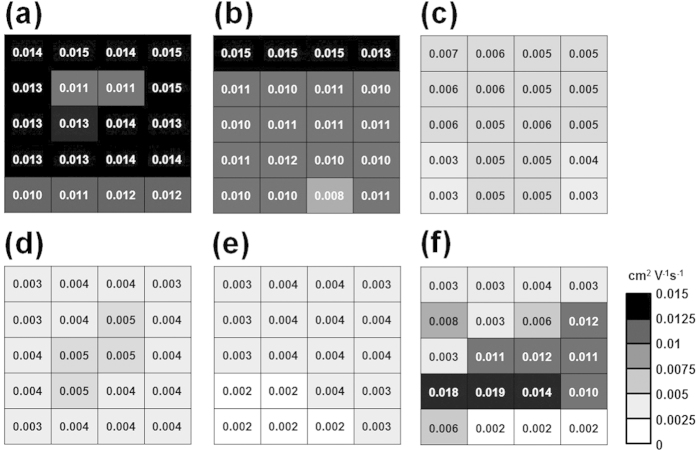
Uniformity of the short-time spin-coating method. The average field-effect mobility in P3HT films that had been spin-cast for various spinning times: (**a**) 3s, (**b**) 5s, (**c**) 10s, (**d**) 30s, and (**e**) 60s. (**f**) The average field-effect mobility in the drop-cast P3HT films. The figures reveal the uniformity of the method. The gradation of colors revealed the field-effect mobility of a unit cell within a one inch square wafer, for each sample.

To demonstrate that our short-time spin-coating method is practically useful, and effective, we compared the reproducibility and uniformity of the charge carrier mobility in P3HT thin films cast for different spinning times (Fig. 5). The FET devices prepared using the drop-cast P3HT films were compared together. We measured the performances of twenty transistor units fabricated on a one inch square wafer. The standard errors of the mobilities measured in the P3HT devices were 11–19% (spin-cast for 3–5s), 17–25% (spin-cast for 10–60s), and 72% (drop-cast)^44^. These electrical property measurements indicated that high-performance uniform polymer thin films could be fabricated using short spinning times.

## Discussion

**Figure 6:**
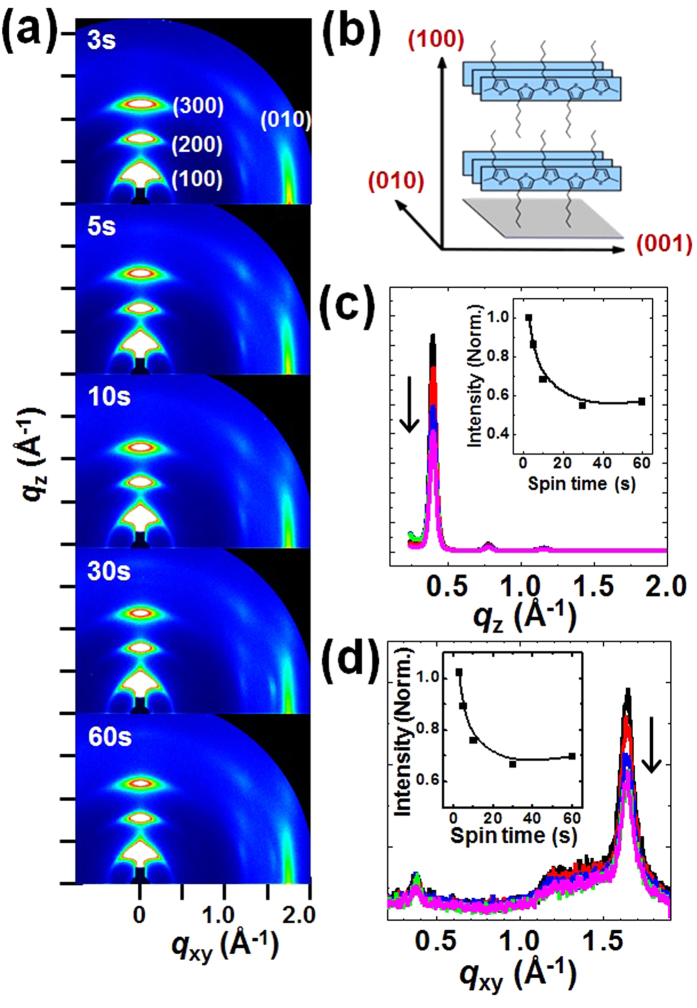
Crystalline characteristics of polythiophene thin films. (**a**) 2D GIXD patterns obtained from the P3HT thin films that had been spin-cast for various spinning times. The spinning time increased from 3 to 60s, in moving from the top toward the bottom figure. (**b**) The schematic illustration provides information about the P3HT crystal structure, with the plane indices. The X-ray intensity 1D profiles obtained from the P3HT thin films along (**c**) the out-of-plane, and (**d**) the in-plane directions. The spinning time increased along the direction of the arrow. The insets show normalized intensities of the (100) or (010) peaks in each scan direction, respectively, as a function of the spinning time.

By varying the spinning time during the spin-coating process, the crystalline structure of P3HT thin films were further characterized in detail using two-dimensional grazing incidence X-ray diffraction (2D GIXD) measurements, which provided information about the long-range crystalline order, thin film texture, cofacial π-π polymer spacing, and stacking orientations relative to the substrate^6^,^45^,^46^,^47^. Figure 6a shows the 2D GIXD patterns obtained from the P3HT films spin-cast over different spinning times from 3 to 60s. All P3HT films displayed strong X-ray reflections due to the (*h*00) and (010) crystal planes along the *q*_z_ and *q*_xy_ axes, respectively, which corresponded to the intermolecular backbone layer and π-π stacking plane distances of 15.7–15.9 Å and 3.8 Å^45^. The crystallographic information is provided in detail in Table 1. These results suggested that most of the P3HT molecules assumed an edge-on chain conformation with side chains oriented in a standing-up configuration on the dielectric substrate, as illustrated in Fig. 6b^48^. The degree of crystallinity in these films depended on spinning time, as revealed in the 1D out-of-plane and in-plane X-ray profiles extracted from the 2D GIXD patterns (Fig. 6c,d). The film thickness values were nearly identical (30 nm), regardless of the spinning time (see Figure S2 in the Supporting Information); therefore, the intensities of the X-ray reflections were directly linked to the degree of crystallinity in the films^49^. Increases in the reflection intensities indicated that the P3HT thin films were more crystalline after shorter spinning times of 3–5s (see the insets in Fig. 6c,d). These results agreed well with the UV-vis, AFM, and device property measurement results reported above.

During spin-coating, the solvent molecules evaporate from the surface of the thin liquid films and migrate quickly toward the film edge under convective flow^27^. If the substrate spinning is stopped before the thin film had completely dries, residual solvent may remain in the polymeric medium to form a highly concentrated gel-like thin film^35^,^36^. The residual solvents would slowly diffuse toward the surface and evaporate through the film surface in a process similar to the slow growth process in operation during drop-casting. Sufficient amounts of residual solvents can mobilize the polymer backbones in the film, which in turn can effectively induce self-organization among the polymer chains to form a highly crystalline structure^4^. Based on this model, we inferred that slow growth processes occurred after spin-coating over brief spinning times, such as 3 or 5s.

The proposed model described above was supported by observed changes in the mean crystallite size as a function of the spinning time. The mean crystallite size in the P3HT films is derived from the correlation length of reflection peaks, which can be estimated using the Scherrer equation given by


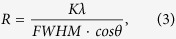


where *K* is the dimensionless Scherrer constant, *λ* is the incident X-ray wavelength, FWHM is the full width at half maximum of the peak, and *θ* is the Bragg angle. The (100) coherent domain sizes in the out-of-plane direction gradually increased from 20.5 to 25.5 nm as the spinning time decreased, suggesting that the stacking defects decreased and the mean crystallite size increased (see Table 1 and Figure S3 in the Supporting Information). This trend in the correlation length values agreed well with the UV-vis spectra and morphology results discussed above. The large mean crystallite size confirmed the presence of a slow growth process in the P3HT films cast over brief spinning times^50^. While the gradual increase in mean crystalline sizes were observed at the out-of-plane direction, the change in the correlation length of the in-plane (010) peak was negligible for all the P3HT films (~12 nm). These results suggested that the enhanced (010) reflection peak and charge transport in the in-plane direction resulted predominantly from an increase in the number of crystallites, rather than growth in the sizes of the crystallites. The FWHM study revealed that brief spinning times strongly reduced disorder in the P3HT film and, in particular, induced the growth of stacked conjugated P3HT segments in the out-of-plane direction.

In addition to studying the effects of the spinning time on the mean crystallite size, we analyzed the paracrystallinity of the P3HT thin films. Structural disorder in an imperfect crystal can be described using the paracrystallinity model as random fluctuations in the lattice spacings^50^,^51^. This disorder is measured quantitatively using the paracrystalline distortion parameter (*g*), defined as the standard deviation of the local static lattice fluctuations normalized by the average value of the lattice spacing^52^. This can be calculated from the slope (=*g*^2^π^2^/*d*; *d* is the domain spacing) of the δ*b* − *h*^2^ plot, where δ*b* is the integral breadth of the diffraction peaks and h is the order of the diffractions. The δ*b* − *h*^2^ curves extracted from 2D GIXD data obtained from the P3HT thin films are plotted in Figure S4 in the Supporting Information, and the crystallographic parameters are listed in Table 1. The calculated paracrystalline distortion parameter values were similar in the P3HT thin films cast for different spinning times, but exceeded the values obtained from the highly crystalline P3HT thin films after post-treatments, such as thermal annealing^52^. These results indicated that the improved performances of devices based on the P3HT films prepared with short spinning times resulted from an increase in the interconnected aggregates on a short length scale, even though these aggregates were small and less ordered than those found in the highly crystalline films.

**Figure 7:**
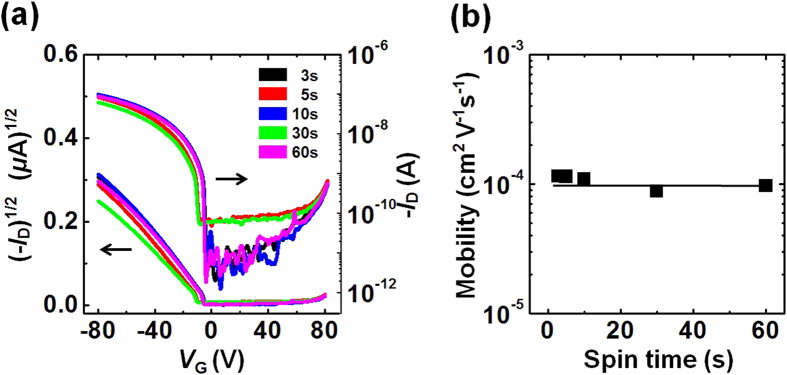
Electrical characteristics of amorphous polymer thin films. (**a**) Transfer characteristics (*I*_D_ − *V*_G_) of FETs (*V*_D_ = −80 V) fabricated from amorphous PTAA thin films that had been spin-cast over various spinning times. (**b**) Field-effect mobilities obtained from the PTAA FETs as a function of the spinning time.

The connection between good performances in devices based on P3HT films cast over short spinning times and the crystalline properties of the P3HT films was further investigated by comparing the results to those obtained from an amorphous semiconducting polymer, polytriarylamine (PTAA)^53^. The amorphous characteristics of the PTAA films were characterized using GIXD measurements (see Figure S5 in the Supporting Information). The FET devices prepared using PTAA films cast over different spinning times exhibited similar charge carrier mobilities (Fig. 7a,b). These results further verified that the slow growth process of polymer crystallites after short spinning times was essential to improving the device performance and interchain ordering in the semicrystalline polymers.

**Figure 8:**
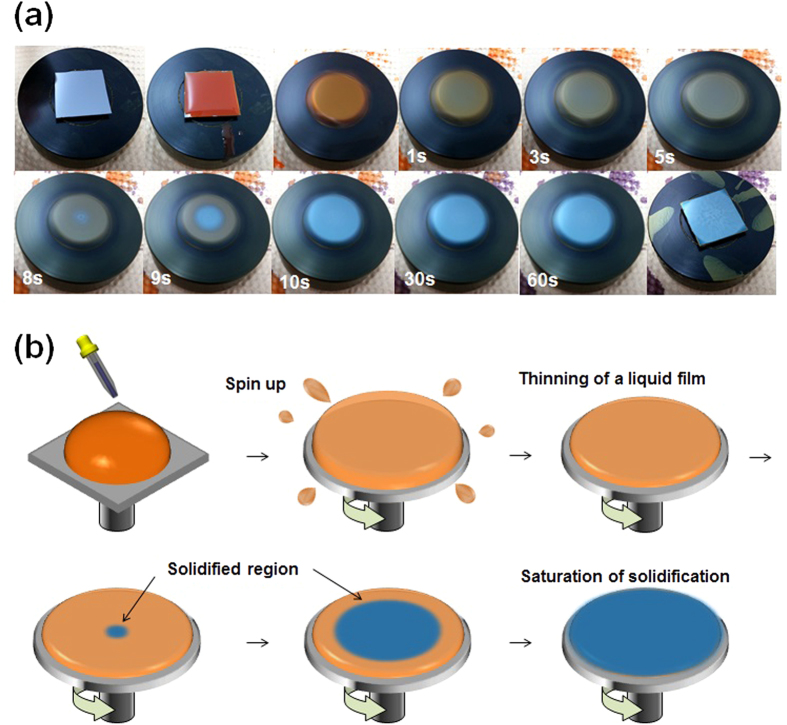
Optical monitoring during spin coating and its schematic illustration. (**a**) Videomicroscopy images of the spin-coating process at specific times after initiation, where the P3HT solution consisted of 1 wt% P3HT in chlorobenzene solvent. (**b**) Schematic diagram showing the spin-coating steps.

The solvent drying behavior during spin-coating is critical for formation of thin films and the development of favorable thin film morphologies and crystalline domains^30^,^49^. The effectiveness of short spin-coating times over a specific range (3–5s) was investigated by monitoring the film drying process using an optical videomicroscopy (Fig. 8a). The recorded video files were carefully analyzed to divide the spin-coating process into several regimes, depending on the color and pattern presented on the thin film (Fig. 8b). As the substrate supporting a P3HT solution droplet began to spin, the color of the substrate changed dramatically. As the substrate spun within the first 1s (the acceleration time of the spin-coater was set to 0s, the minimum value), the thickness of the solution droplet decreased dramatically, rending the deep orange color of the solution more translucent. The liquid film continued to thin over 5s. Between 5 and 8s, the color at the center of the substrate began to transition to the color of the final thin film, indicating the onset of film formation, because the UV-vis absorption band obtained from P3HT in the solution state differs from that obtained from P3HT in the solid state (Figure S1 in the Supporting Information). Ten seconds after the substrate spinning was initiated, the color of the whole film remained unchanged, indicating that the residual solvent was almost completely removed and the P3HT solidification was completed. We define this time as the complete solidification time (*t*_s_). The value of *t*_s_ = ~10s, as observed in our study, was similar with the value from the value revealed from the *in-situ* GIXD and UV-vis studies during spin-coating, confirming the reliability of the experimental method^54^,^55^. If the spinning process is stopped prior to complete drying or solidification of the P3HT films, the residual CB molecules can mobilize the P3HT chains and will effectively induce self-organization through a subsequent slow growth process. Optical monitoring experiments revealed that the complete solidification process required 10s for a P3HT/CB solution, which explains why a short spinning time of 3 to 5s was specifically effective in improving the device performance.

**Figure 9:**
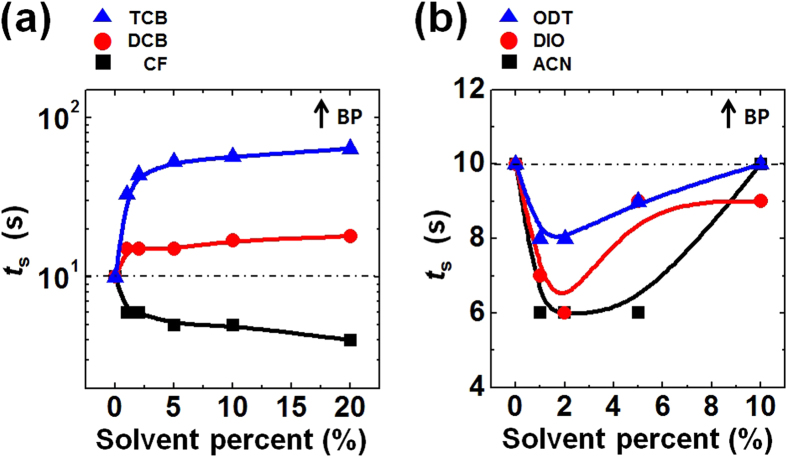
Summary of optical monitoring analysis with processing additives. Complete solidification time (*t*_s_) during spin-coating as a function of the additive volume ratios of the P3HT solutions: (**a**) good solvent additives and (**b**) non-solvent additives, respectively. The boiling point of the additive increased along the direction of the arrow.

A deeper understanding of the film drying behavior during spin-coating was sought by optically monitoring samples prepared with various processing additives. The solvent additives can be categorized as good or poor solvents (non-solvents) with different boiling points (BPs), depending on their ability to dissolve P3HT^56^. The additive solvents were gradually added to the P3HT/CB solutions in various volume ratios with respect to CB (the main solvent) to a total concentration of 1 wt%. The optical changes that took place during the spin-coating of these solutions are visualized in Figures S6–S11 in the Supporting Information. In contrast with the P3HT/CB system exhibiting the clear onset of solidification, it was difficult to unambiguously define the onset point in the additive processed systems due to the different evaporation rates in the mixed solvent. Instead, we observed variations in *t*_s_ as a function of the mixed volume ratios, as observed in Fig. 9a,b. The high-BP good solvents 1,2-dichlorobenzene (DCB, BP of 181 °C) and 1,2,4-trichlorobenzene (TCB, BP of 214 °C) produced an increase in *t*_s_ as the volume ratio of the additive increased (Fig. 9a). By contrast, the addition of chloroform (CF, BP of 61 °C), a low-BP good solvent, gradually reduced *t*_s_. These results demonstrated that the process associated with drying a solution comprising good solvents may be controlled by both the volume ratio and the BPs of the additives^56^,^57^,^58^. Because the BPs of DCB and TCB exceed the BP of CB, the evaporation rate of CB molecules at the surface of a liquid is much faster than the evaporation rates of DCB or TCB^58^. Therefore, the film drying behavior may be mainly controlled by DCB or TCB molecules rather than CB. By contrast, CF molecules tend to evaporate more rapidly than CB molecules in a mixture of CF and CB so that the completion of the drying process is determined by the amount of CB in the solution. We could conclude that *t*_s_ varied in correlation with the additive BP in the good solvent additive system.

Three types of non-solvent were used in non-solvent additive systems: acetonitrile (ACN, BP of 82 °C), 1,8-diiodooctane (DIO, BP of 168 °C), and 1,8-octanedithiol (ODT, BP of 270 °C). The values of *t*_s_ in the good solvent additive system varied in relation with the BPs, whereas the non-solvent additives generally reduced *t*_s_ in relation to the volume ratio (Fig. 9b). Based on the our previous studies, the addition of small amounts of non-solvent additives transforms P3HT in solution from a random coil to an ordered aggregate^16^,^59^. The degree of precursor ordering in the solutions significantly increases the crystallinity of the P3HT thin films^60^. A reduction in *t*_s_ as a result of the addition of a non-solvent additive suggested that the ordered P3HT aggregates accelerated the solidification of P3HT, possibly resulting from the lower energy barrier of the seeded growth^61^. As the amount of non-solvent additives increased, the values of *t*_s_ increased again, possibly due to the high BPs of the additives, similar as observed in the presence of the good solvent additives.

**Figure 10:**
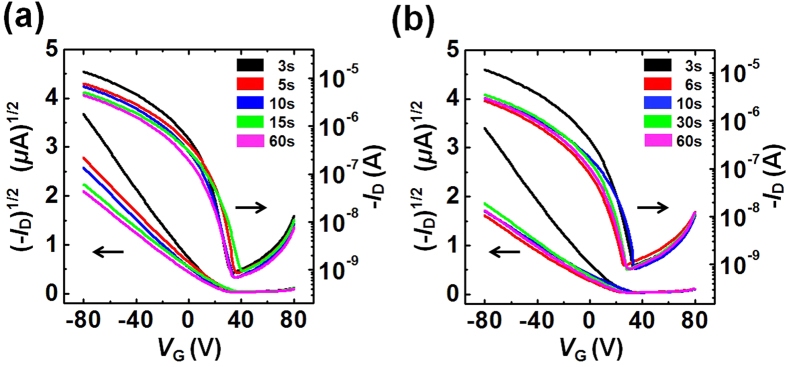
Electrical characteristics of polythiophene films processed with additives. Transfer characteristics (*I*_D_ − *V*_G_) of FETs (*V*_D_ = −80 V) fabricated using P3HT thin films that had been spin-cast for various spinning times, wherein the P3HT thin films were spin-coated using 2 vol% solvent additives: (**a**) 1,2-dichlorobenzene (DCB) or (**b**) acetonitrile (ACN).

**Figure 11:**
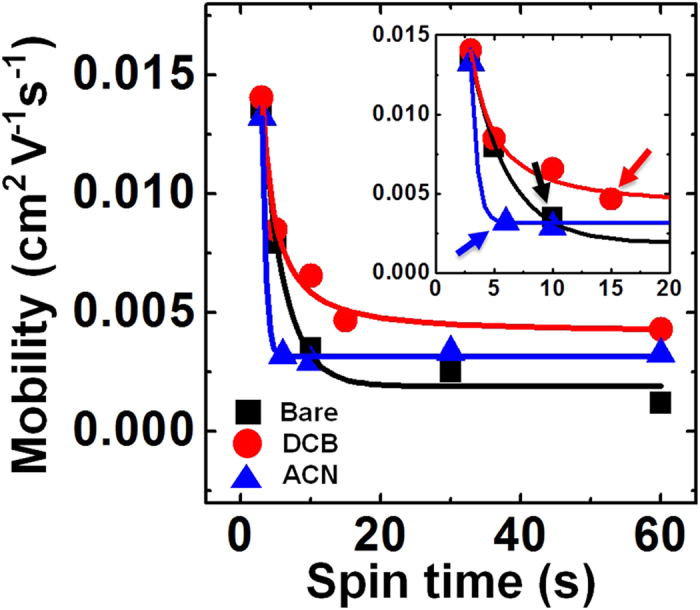
Summary of P3HT FETs processed with additives. Field-effect mobilities obtained from the P3HT FETs as a function of the spinning time. BARE denotes the P3HT FETs prepared without using solvent additives. The inset highlights the difference between the onset point marking the beginning of charge carrier mobility reduction. The arrows provide guides to highlight the saturation behavior in the mobility values.

The effects of the additives on the solidification time were examined by measuring the performances of FETs prepared using additive-processed P3HT films that had been spin-cast for various spinning times (3–60s). Figure 10a,b show the transfer characteristics of the P3HT FETs prepared using P3HT films that had been processed using 2 vol% DCB or ACN additives, respectively. Interestingly, the maximum mobility values were observed in P3HT films cast over 3s, with or without the additives, and the mobility values were similar (Fig. 11). Apparently, the charge carrier mobility in these devices tended to decrease for longer spinning times, but it should be emphasized that the saturated mobility values remained higher than those of devices prepared without additives. These results suggested that the presence of the additives in general enhanced the crystallinity of the polymeric film, although the nature of the crystallization behaviors depended on the specific additive used^56^,^58^. The mobility enhancement observed in P3HT films that had been spin-cast for 60s was similar for both additives, although the trend in the mobility decrease differed considerably (see the inset shown in Fig. 11). The mobility decrease continued until reaching a spinning time of 15s for the DCB additive. By contrast, the mobility values abruptly reached saturation in the presence of the ACN additive over a spinning time of 5s. These differences between the trends in the mobility values agreed well with previous results collected in the optical monitoring experiments, as shown in Fig. 9. A good solvent additive with a high BP delayed the complete solidification of the P3HT film, which extended the effective range of the spinning time over which the slow growth process occurred after the spinning had been intentionally stopped. By contrast, the non-solvent additives induced ordered structure formation among the precursors in the solution^62^, which shortened the solidification time of the P3HT film. These results suggested that the aggregated state minimized unfavorable interactions among the poorly soluble P3HT chains and the non-solvent ACN and maximized the favorable stacking interactions among the P3HT chains through π-π stacking^57^. Unfavorable contacts between the P3HT molecules and the solvent allowed for rapid evaporation and growth among the ordered polymer crystallites during the spin-coating process. The values of *t*_s_ that were reduced by the presence of the non-solvent additives, which decreased the solubility of P3HT, suggested that the ordered P3HT aggregates accelerated the solidification of P3HT, possibly by minimizing the formation of unfavorable contacts.

## Conclusions

In conclusion, the field-effect mobilities of crystalline polymer semiconductors could be enhanced by spin-coating a semiconductor solution over a short period of only a few seconds. The lamellar stacking and π-π stacking interactions among the P3HT thin films were improved by the slow growth process resulting from the residual solvent after a brief spin-coating time. Highly crystalline, uniform, and reproducible polymer thin films could be prepared using this method, and no post-treatments were required. We demonstrated that the volume, boiling point, and dissolving power of the added solvents dramatically influenced the P3HT film solidification process as well as the electrical properties of the resulting P3HT films. This simple method for enhancing molecular ordering is clearly useful to the development of robust and practical polymer devices for a wide range of commercial applications.

## Methods

### Preparation of Polythiophene Thin Films and FET Devices

Poly(3-hexylthiophene) (P3HT), obtained from Rieke Metals, Inc. (regioregularity ~95%, molecular weight, *M*_w_ = 20 − 30 kDa) was used as received without further purification. Highly doped Si was used as a gate electrode as well as a substrate. A thermally grown 300 nm thick SiO_2_ layer was employed as a gate dielectric (capacitance = 10.8 nF cm^−2^). Substrates were cleaned in acetone and ethanol for 30 min using an ultrasonicator, followed by drying under a N_2_ stream prior to use. Hexamethyldisilazane (HMDS) (Aldrich) was used as an organic interlayer material between the organic active material and the dielectric layer and was applied to the SiO_2_ substrate via spin-casting. Chloroform, chlorobenzene, 1,2-dichlorobenzene, 1,2,4-trichlorobenzene, acetonitrile, 1,8-diiodoctane, and 1,8-octanedithiol were used as received from Aldrich. The P3HT chlorobenzene solution was prepared in sealed vials by dissolution at 40 °C to protect the solution from evaporation. The warm P3HT solution was cooled to room temperature. The additive solvents were gradually introduced in various volume ratios with respect to chlorobenzene into the P3HT solutions to a total concentration of 1 wt%. The solutions were then stirred overnight at 50 °C. The PTAA chlorobenzene solution (0.5 wt%) was dissolved at 40 °C. P3HT or PTAA thin films were spin-coated at 1500 rpm for various spinning times (Spin-1200D, Midas). P3HT (or PTAA)-based OFETs were formed by evaporating gold through a shadow mask (channel length = 100 μm and channel width = 2000 μm). Identical P3HT films were fabricated on transparent glass substrates in place of Si substrates in preparation for the UV-Vis absorption measurements.

### Characterization

UV-Vis absorption spectra were acquired using a UV-Vis spectrophotometer (CARY-5000, Varian). The thickness values of the cast P3HT films were measured using an ellipsometer (J. A. Woollam Co. Inc.). The film morphologies were characterized by atomic force microscopy (AFM, Multimode 8, Digital Instruments). Grazing incidence X-ray diffraction (GIXD) studies were performed at the 3C, 5A, 9A, and 9C beam lines of the Pohang Accelerator Laboratory, Korea. The electrical performances of the OFETs were characterized using a semiconductor analyzer (Keithley 4200) at room temperature. The field-effect mobility (*μ*_FET_) and threshold voltage (*V*_T_) were estimated in the saturation regime (*V*_D_ = −80 V) according to the equation^10^:


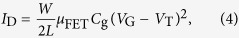


where *I*_D_ is the drain current, *C*_g_ is the capacitance of the gate dielectric, and *V*_G_ is the gate–source voltage.

## Additional Information

**How to cite this article**: Na, J. Y. *et al.* Understanding Solidification of Polythiophene Thin Films during Spin-Coating: Effects of Spin-Coating Time and Processing Additives. *Sci. Rep.* **5**, 13288; doi: 10.1038/srep13288 (2015).

## Supplementary Material

Supplementary Information

## Figures and Tables

**Table 1:** Crystallographic information of polythiophene thin films.

Crystallographic parameters		3s^a^	5s	10s	30s	60s
lamella stack (out-of-plane)	*q* (Å^−1^)	0.39552	0.39896	0.39708	0.3972	0.39761
	*d*-spacing (Å)	15.9	15.7	15.8	15.8	15.8
	FWHM (Å^−1^)	0.04444	0.05006	0.05383	0.05546	0.05525
	Correlation length (Å)	254.6	226.1	210.2	204.1	204.8
	*g* (%)^b^	10.97	10.98	10.68	10.52	10.79
	*N*_avg_^diff,^ c	8.42	8.41	8.88	9.15	8.70
π - π stack (in-plane)	*q* (Å^−1^)	1.63777	1.64014	1.64019	1.63916	1.64052
	*d*-spacing (Å)	3.8	3.8	3.8	3.8	3.8
	FWHM (Å^−1^)	0.09501	0.08971	0.0896	0.09272	0.09334
	Correlation length (Å)	120.3	127.4	127.6	123.3	122.5

P3HT thin films had been cast for various spinning times. The correlation length was determined from the full width at half maximum (FWHM) value of the X-ray diffraction pattern using the Scherrer equation.

^a^Spin-coating time applied to the P3HT thin films prepared on HMDS-treated SiO_2_/Si substrates.

^b^Paracrystalline distortion parameters.

^c^Average number of diffraction planes.
