# Supplementary material for: Understanding Solidification of Polythiophene Thin Films during Spin-Coating: Effects of Spin-Coating Time and Processing Additives
Source: Sci Rep. 2015 Aug 24;5:13288. doi: 10.1038/srep13288 (PMC4547395; doi:10.1038/srep13288)
Supplement: Supplementary Information [file srep13288-s1.doc]

**Supplementary Information**

**Understanding Solidification of Polythiophene Thin Films during Spin Coating: Effects of Spin-Coating Time and Processing Additives**

*Jin Yeong Na*^1,†^, *Boseok Kang*^2,†^, *Dong Hun Sin*^2^, *Kilwon Cho*^2^,* *and Yeong Don Park*^1^,*

^1^ Department of Energy and Chemical Engineering, Incheon National University, Incheon 406-772, Korea

^2^ Department of Chemical Engineering, Pohang University of Science and Technology

Pohang 790-784, Korea.

*E-mail: ydpark@inu.ac.kr (ydp), and kwcho@postech.ac.kr (kc)

† These authors contributed equally to this work.


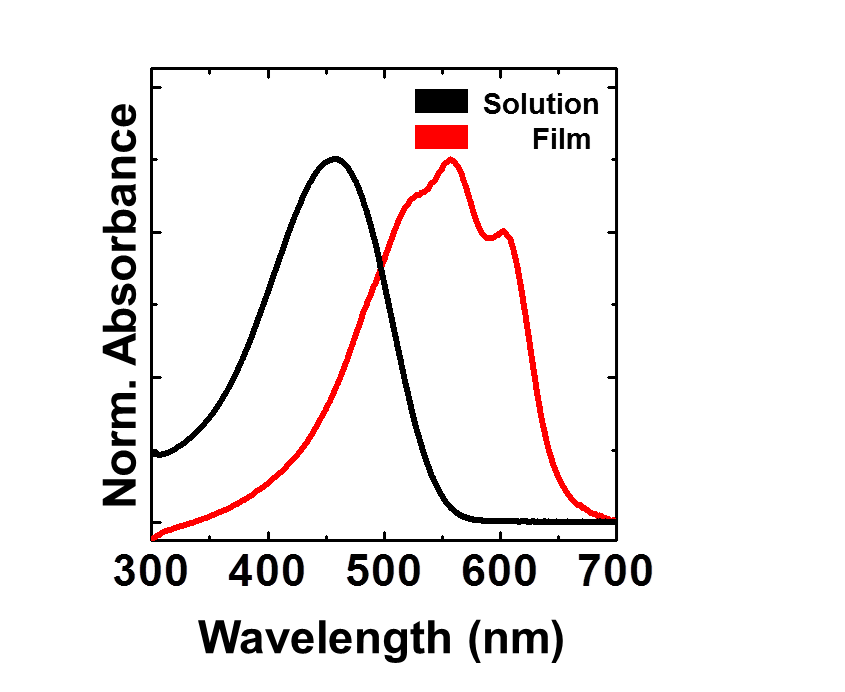


**Figure S1.** Normalized absorption spectra of P3HT in solution or in the thin film state.


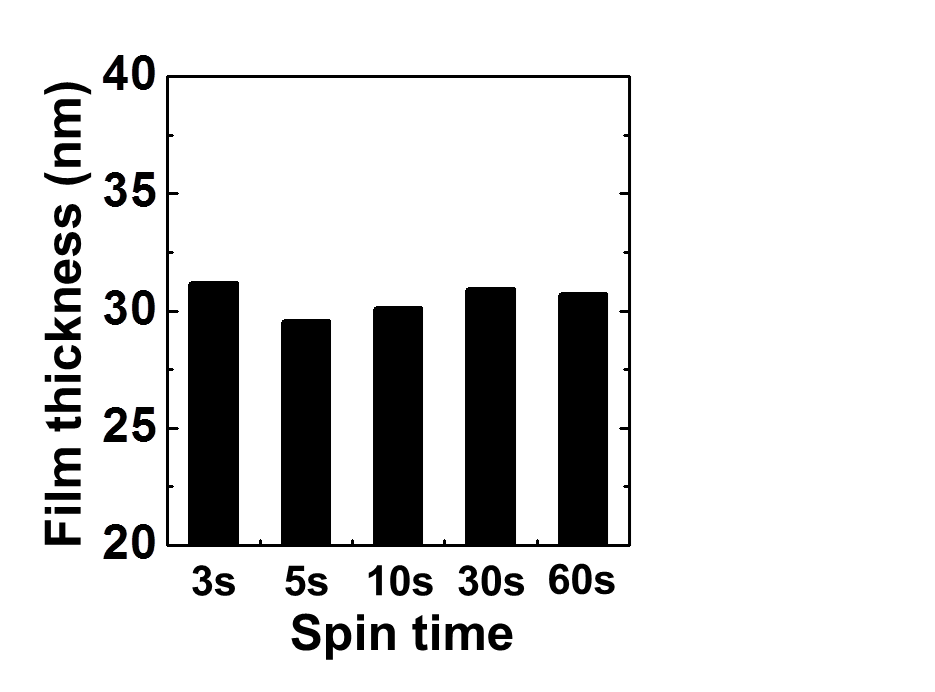


**Figure S2.** Film thickness values of the P3HT thin films that had been spin-cast over the indicated spinning times.


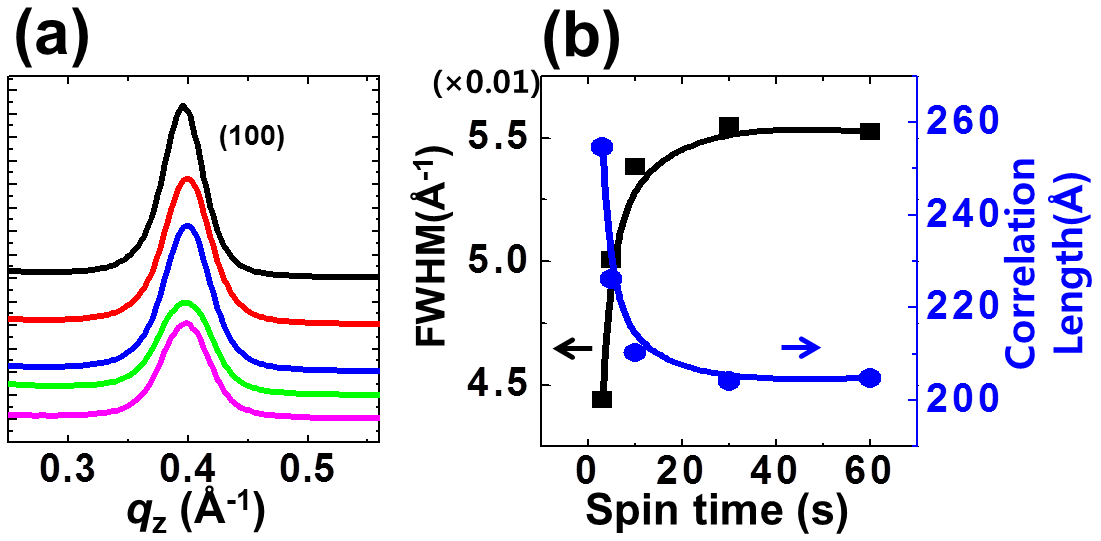


**Figure S3.** (a) Magnified (100) X-ray profiles obtained from P3HT thin films along the out-of-plane direction. (b) FWHM of the X-ray diffraction pattern (left axis) and the correlation length (right axis) calculated from the Scherrer equation, for various spinning times.


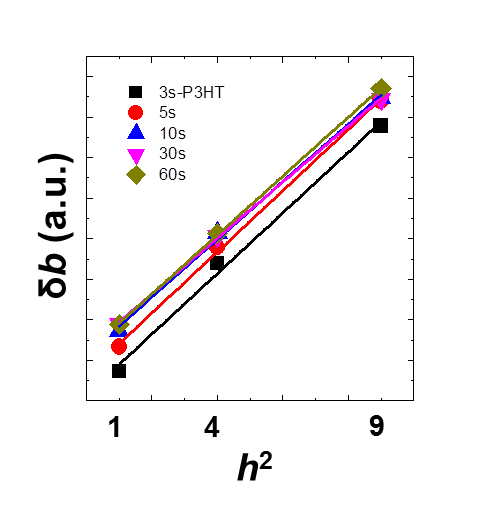


**Figure S4.** δ*b*-*h*^2^ plots extracted from the 2D GIXD analysis, where δ*b* indicates the integral width of each diffraction peak and h indicates the order of the diffraction peaks.


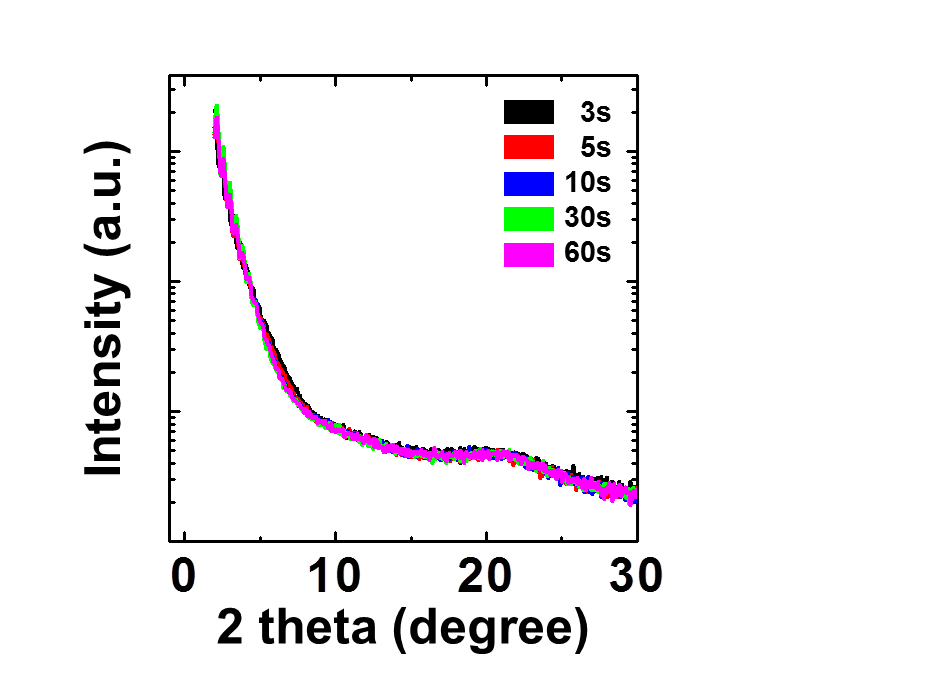


**Figure S5.** Out-of-plane GIXD intensities as a function of the scattering angle 2θ for PTAA thin films prepared on SiO_2_/Si substrates and spin-cast over various spin times.


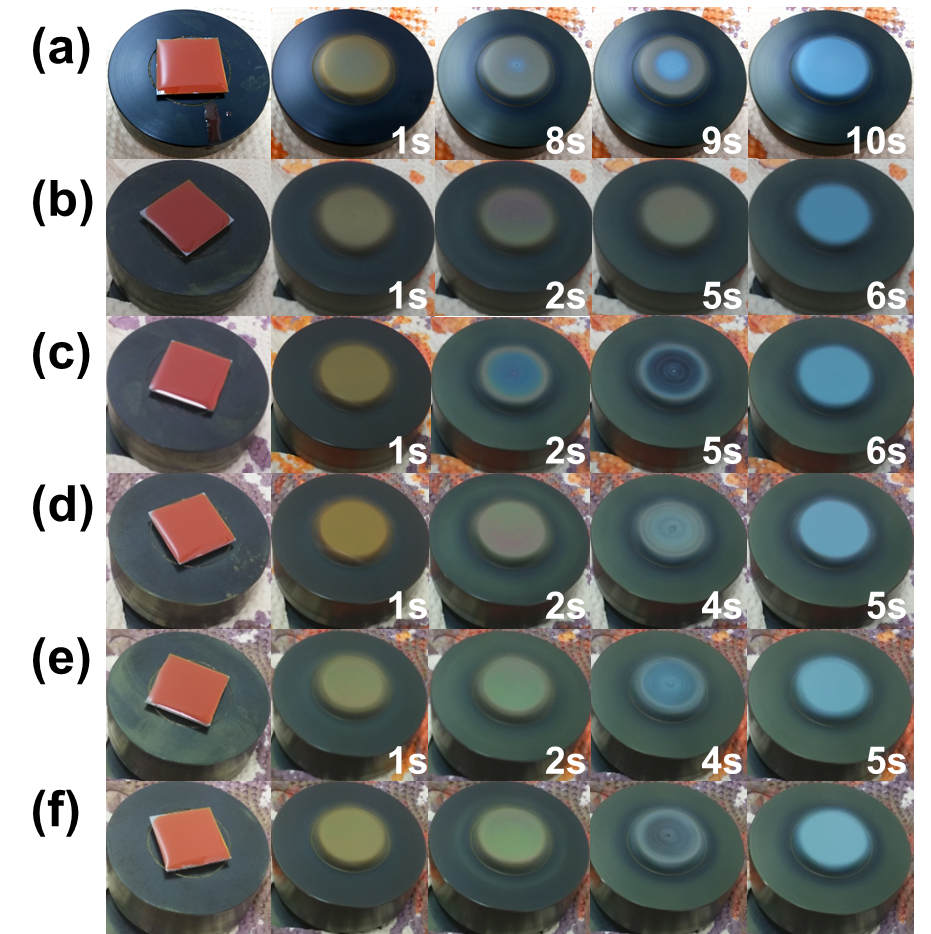


**Figure S6.** Videomicroscopy images of the spin-coating process at specific times after initiation. The additive solvent, chloroform, was gradually introduced in various volume ratios with respect to chlorobenzene into the P3HT solutions to a total concentration of 1 wt%: (a) 0%, (b) 1%, (c) 2%, (d) 5%, (e) 10%, and (f) 20%.


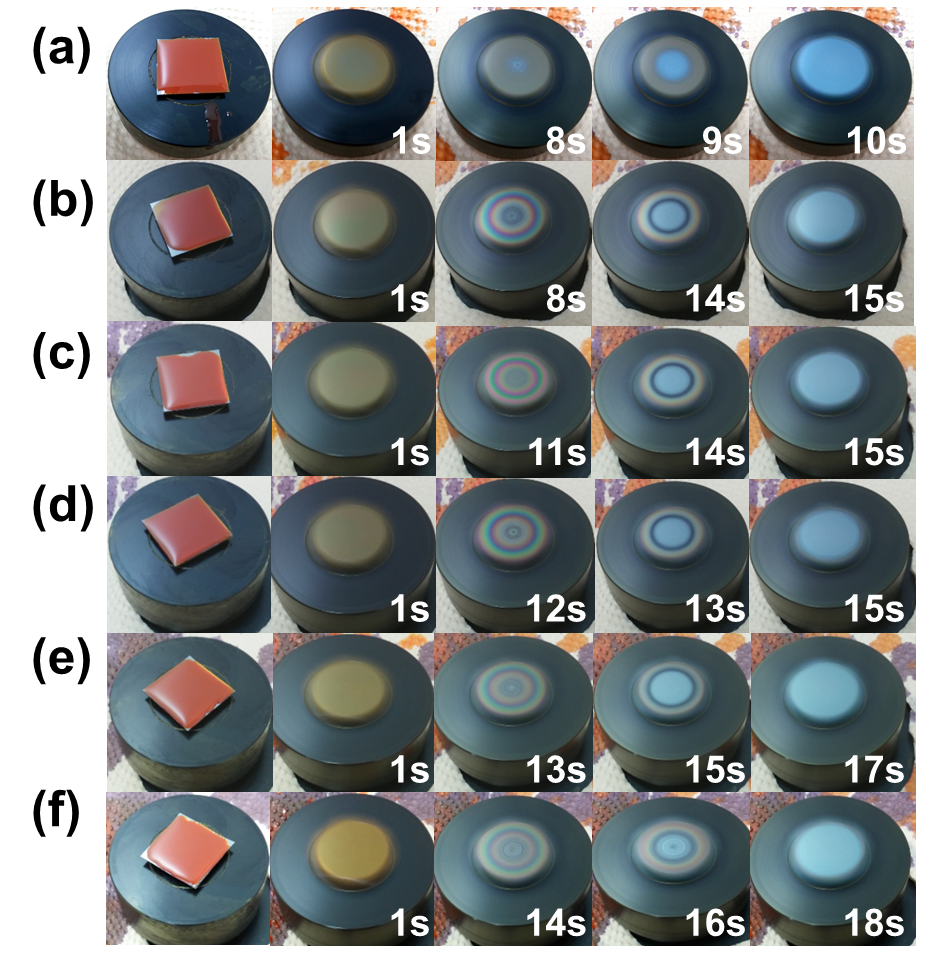


**Figure S7.** Videomicroscopy images of the spin-coating process at specific times after initiation. The additive solvent, 1,2-dichlorobenzene (DCB), was gradually introduced in various volume ratios with respect to chlorobenzene into the P3HT solutions to a total concentration of 1 wt%: (a) 0%, (b) 1%, (c) 2%, (d) 5%, (e) 10%, and (f) 20%.


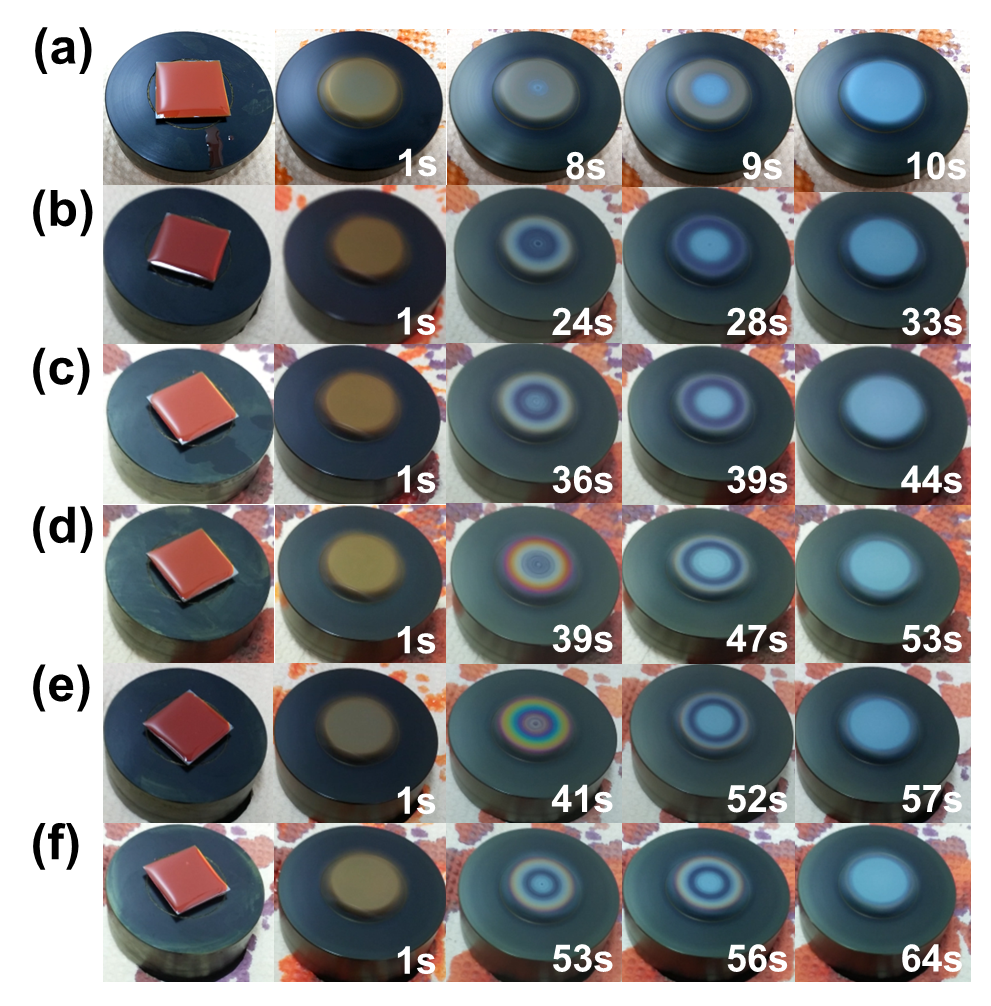


**Figure S8.** Videomicroscopy images of the spin-coating process at specific times after initiation. The additive solvent, 1,2,4-trichlorobenzene (TCB), was gradually introduced in various volume ratios with respect to chlorobenzene into the P3HT solutions to a total concentration of 1 wt%: (a) 0%, (b) 1%, (c) 2%, (d) 5%, (e) 10%, and (f) 20%.


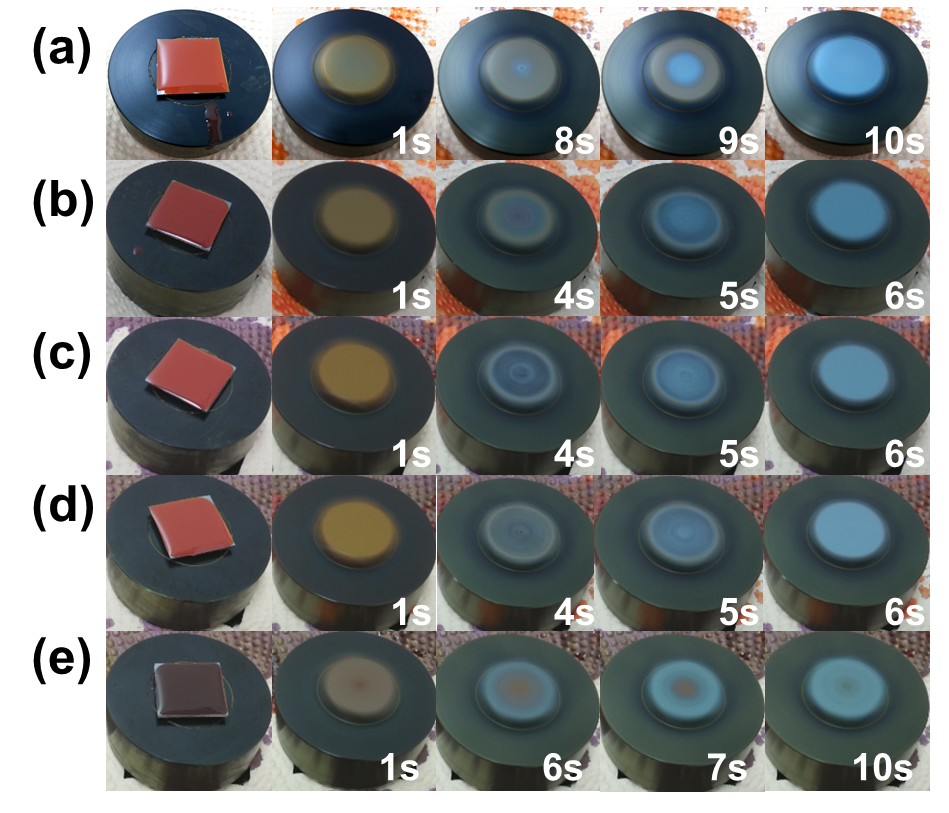


**Figure S9.** Videomicroscopy images of the spin-coating process at specific times after initiation. The additive solvent, acetonitrile (ACN), was gradually introduced in various volume ratios with respect to chlorobenzene into the P3HT solutions to a total concentration of 1 wt%: (a) 0%, (b) 1%, (c) 2%, (d) 5%, and (e) 10%.


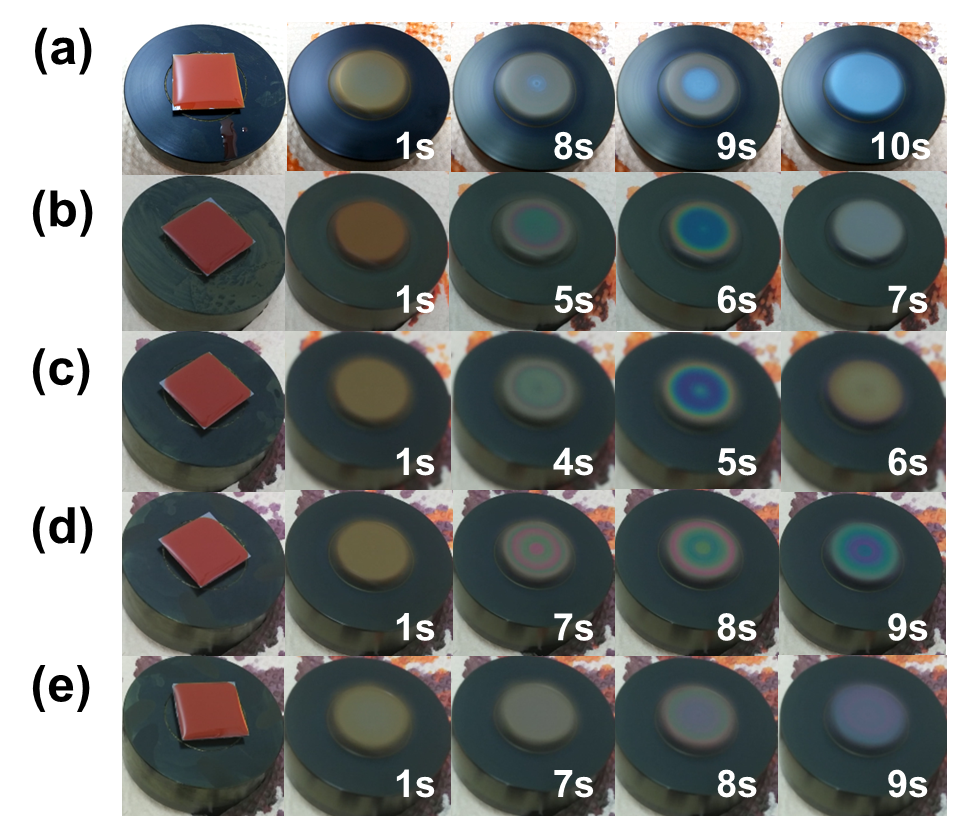


**Figure S10.** Videomicroscopy images of the spin-coating process at specific times after initiation. The additive solvent, 1,8-diiodooctane (DIO), was gradually introduced in various volume ratios with respect to chlorobenzene into the P3HT solutions to a total concentration of 1 wt%: (a) 0%, (b) 1%, (c) 2%, (d) 5%, and (e) 10%.


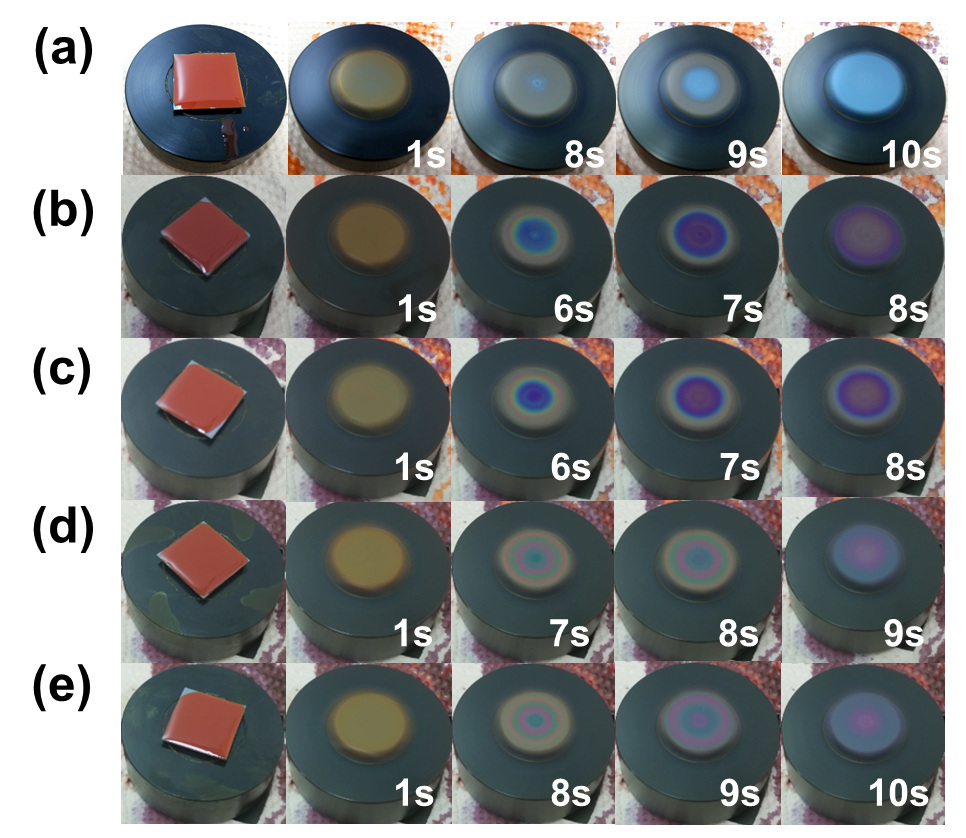


**Figure S11.** Videomicroscopy images of the spin-coating process at specific times after initiation. The additive solvent, 1,8-octanedithiol (ODT), was gradually introduced in various volume ratios with respect to chlorobenzene into the P3HT solutions to a total concentration of 1 wt%: (a) 0%, (b) 1%, (c) 2%, (d) 5%, and (e) 10%.
